# Maximising recruitment to a randomised controlled trial for chronic rhinosinusitis using qualitative research methods: the MACRO conversation study

**DOI:** 10.1186/s13063-020-04993-w

**Published:** 2021-01-13

**Authors:** Clare McDermott, Jane Vennik, Carl Philpott, Steffi le Conte, Mike Thomas, Caroline Eyles, Paul Little, Helen Blackshaw, Anne Schilder, Claire Hopkins

**Affiliations:** 1 Primary Care and Populations Sciences, Faculty of Medicine, University of Southampton, Southampton, UK; 2 Norwich Medical School, University of East Anglia, Norwich, UK; 3 James Paget University Hospital NHS Foundation Trust, Great Yarmouth, UK; 4 Surgical Interventional Trials Unit, University of Oxford, Oxford, UK; 5 evidENT, Ear Institute, University College London, London, UK; 6 Guy’s and St. Thomas, NHS Foundation Trust, London, UK

**Keywords:** Randomised controlled trial, Recruitment, Chronic rhinosinusitis, Qualitative research, Thematic analysis

## Abstract

**Background:**

Randomised controlled trials (RCTs) are considered the ‘gold standard’ of medical evidence; however, recruitment can be challenging. The MACRO trial is a NIHR-funded RCT for chronic rhinosinusitis (CRS) addressing the challenge of comparing surgery, antibiotics and placebo. The embedded MACRO conversation study (MCS) used qualitative research techniques pioneered by the University of Bristol QuinteT team to explore recruitment issues during the pilot phase, to maximise recruitment in the main trial.

**Methods:**

Setting: Five outpatient Ear Nose and Throat (ENT) departments recruiting for the pilot phase of the MACRO trial (ISRCTN Number: 36962030, prospectively registered 17 October 2018). We conducted a thematic analysis of telephone interviews with 18 recruiters and 19 patients and 61 audio-recordings of recruitment conversations. We reviewed screening and recruitment data and mapped patient pathways at participating sites. We presented preliminary findings to individual site teams. Group discussions enabled further exploration of issues, evolving strategies and potential solutions. Findings were reported back to the funder and used together with recruitment data to justify progression to the main trial.

**Results:**

Recruitment in the MACRO pilot trial began slowly but accelerated in time to progress successfully to the main trial. Research nurse involvement was pivotal to successful recruitment. Engaging the wider network of clinical colleagues emerged as an important factor, ensuring the patient pathway through primary and secondary care did not inadvertently affect trial eligibility. The most common reason for patients declining participation was treatment preference. Good patient-clinician relationships engendered trust and supported patient decision-making. Overall, trial involvement appeared clearly presented by recruiters, possibly influenced by pre-trial training. The weakest area of understanding for patients appeared to be trial medications. A clear presentation of medical and surgical treatment options, together with checking patient understanding, had the potential to allay patient concerns.

**Conclusion:**

The MACRO conversation study contributed to the learning process of optimising recruitment by helping to identify and address recruitment issues. Although some issues were trial-specific, others have applicability to many clinical trial situations. Using qualitative research techniques to identify/explore barriers and facilitators to recruitment may be valuable during the pilot phase of many RCTs including those with complex designs.

## Background

Randomised controlled trials (RCTs) are widely considered the ‘gold standard’ of medical evidence [1]. However, recruitment to RCTs can be problematic. In 2017, Walters et al. reviewed 151 trials funded by the UK Health Technology Assessment Programme (HTA) and found that only 56% of the RCTs reached their original recruitment target sample size, although 79% reached 80% of their target [2]. Similar results have been found by other reviews [3]. Poor recruitment can lead to a trial being statistically underpowered, increasing the risk of type II errors. Furthermore, if a trial has to be abandoned, wasted resources include not only funding but the time and effort contributed by clinicians, researchers and patients [4].

Within this context, maximising recruitment is a priority for all trialists. The use of qualitative research techniques to understand barriers and optimise recruitment has been pioneered by the Qualitative Research Integrated within Trials (QuinteT) team from the University of Bristol [5]. This approach, which Donovan and colleagues term ‘QuinteT Recruitment Intervention’ (QRI) involves two stages [6]. In phase I, data is gathered from recruiter and patient interviews, audio-recordings of recruitment conversations, screening logs and recruitment pathways for each research site. Data from all sources is then triangulated to identify barriers and facilitators to recruitment [7]. In phase 2, initial findings are presented and discussed with the Chief Investigator, Trial Management Group and Clinical Trials Unit so that an ‘action plan’ can be agreed and implemented to address identified barriers to recruitment. The approach has been implemented within a number of diverse trials as a means of enhancing recruitment [8] leading to increasing interest among trialists in its use, especially for clinical trials where recruitment is anticipated to be complex or challenging.

Chronic rhinosinusitis affects up to 11% of the European population [9], making it one of the most common chronic conditions in the Western world. The condition is characterised by inflammation of the nose and paranasal sinuses for 12 weeks or more and is commonly divided into two groups determined by the presence or absence of nasal polyps [10]. The MACRO programme was established with the aim of determining the best management for adult chronic rhinosinusitis (CRS) [11]. A key component of this NIHR funded programme is the MACRO randomised controlled trial (RCT) comparing endoscopic sinus surgery, antibiotic treatment and standard medical care (trial interventions and other aspects of trial design are summarised in Table 1: Trial design). To address the challenges and uncertainties of evaluating medical and surgical treatments in the same trial, an expert panel consensus process was used to optimise trial design [12]. The panel reviewed current evidence and mixed method data collected in the development phase of the MACRO programme and agreed a design that was considered achievable in terms of recruitment, whilst addressing the aims of establishing best management of patients with CRS. Prior work by the MACRO team at two of the trial sites had also established acceptability of the trial processes including the outcome measures utilised and the duration of trial visits [13]. The key recruiters for the MACRO trial are ENT surgeons, ENT physicians and specialist ENT nurses working as research nurses for the trial.

**Table 1:** Trial design

**The MACRO trial** [11] **Design:** Three-arm parallel-group RCT. **Setting**: Outpatient Ear, Nose and Throat (ENT) departments in tertiary care. **Participants**: Adults with uncomplicated Chronic Rhinosinusitis with or without nasal polyps. **Interventions**: Participants randomised to receive: (1) intranasal medication plus ESS, (2) intranasal medication plus clarithromycin (250 mg) or (3) intranasal medication plus a placebo. The placebo consisted of an inert substance encapsulated in a red capsule. In order to achieve double blinding, the antibiotic clarithromycin was encapsulated in identical red capsules and all packaging was identical. Intranasal medication (current standard medical care) is defined as a spray or drops of intranasal corticosteroids and saline irrigations. **Primary outcome measure**: change at 6 months in the the SNOT-22 questionnaire, which assesses disease-specific health-related quality of life. **Study sample size**: pilot phase *n* = 3. Main trial *n* = 600.	

A pilot phase provided the opportunity to determine whether recruitment processes and targets were feasible and achievable prior to progression to a main trial. The MACRO conversation study (MCS) modelled on the Bristol QRI approach [6] nested in the pilot phase, aimed to identify and understand recruitment issues to maximise recruitment processes prior to the main trial. Whilst recruitment issues have been explored in other trials [8, 14, 15], we were particularly interested in issues specific to the MACRO trial, with the complexities of comparing medical and surgical treatment arms. With a target of 600 patients to be recruited over 2.5 years, MACRO represents the largest ENT trial undertaken to date in the UK [11, 16].

## Methods

A total of six outpatient ENT Departments (sites) took part in the pilot phase of the MACRO trial between December 2018 and June 2019. Recruiters were defined as medical staff who spoke with potential patients about the MACRO trial and included surgeons, consultant physicians, research nurses and research assistants. Of these, surgeons and physicians would normally conduct the main recruitment conversation where the trial design was presented and explained to the patient, with research nurses or research assistants carrying out further conversations with patients to take time to go through the patient information sheet and any queries in more detail.

Trial specific training materials were prepared based on trial team experience, qualitative work from the development stage of MACRO [17, 18], existing published research and published investigator training materials from the QuinteT team [19–23]. Particular emphasis was placed on general recruitment challenges, maintaining equipoise, communicating the concept of randomisation and how to manage perceived patient preference in particular reference to the MACRO trial (see Table 2). These training materials were presented at an investigator meeting (April 2018, London) and also as part of each site initiation visit. All recruiters received a copy of the training materials and copies were emailed to any recruiter unable to attend the meeting.

**Table 2:** Examples from training materials for recruiters to the MACRO trial (informed by training materials for recruitment to trials developed by the QuinteT Team [19])

**The MACRO Guidance for recruiters was structured under the following headings:** ‘Starting the appointment’, ‘Explaining the study design’, ‘Explaining timing for the study and follow up’ and ‘Closing the appointment’.	
**Two examples from the training notes are given below.**	
**‘Explaining the study design’: examples of guidance**	
• ‘Ask patients to **keep an open mind** until you have presented all the information	
• Present **balanced information** about all treatment options. It is best to call the arms **‘treatment option 1’**, ‘**treatment option 2’** and **‘treatment option 3’**. Try to avoid the use of ***standard*** or ***experimental*** treatment	
• When explaining the study design, you may find it useful to **draw the treatment options** on a piece of paper or use a simple pre-prepared diagram like that found in the MACRO Patient Information Sheet *(training materials replicate suitable diagram here).*	
•Gently explore any patient **preferences** to **uncover** and **challenge any misunderstandings’**	
**Explaining treatment allocation/randomisation: examples of guidance**	
• ‘Explain to patients that they will be allocated to a treatment option by a process called **randomisation**	
• Explain that they will have an **equal chance** of receiving each of the three treatment options, but **neither the patient nor the doctor can choose the treatment option**	
• Explain that randomisation is used to ensure the groups can be **compared fairly** by making sure that each group is similar-	
• **Avoid** using terms such a **‘toss of a coin’** or ‘**decided by computer’**. You may want to say something like this:	
‘If you agree to take part in the MACRO study you will be allocated to one of three treatment options through a process called randomisation. This means that you will be assigned to one of the options by chance and neither you nor your doctor can choose. This is so that the options can be compared fairly-each group will contain similar numbers and be similar in all other ways. You will have an equal chance of getting each of the treatment options.’	

In order to explore the recruitment issues, we collected and analysed four data sources: (1) audio-recordings of recruitment conversations between recruiters and patients, (2) semi-structured telephone interviews with recruited patients, (3) semi-structured telephone interviews with recruiters, and (4) site screening logs completed by recruiters. In addition, recruiters were asked during their interview to describe the recruitment pathway for their site, so that this information could be used by the researchers to understand and map the stages and processes involved. Face-to-face recruitment consultations involved one or more recruiters and the patient at a clinic appointment. Telephone conversations involved patients and research nurses only. All recruiting sites were asked to record all recruitment conversations where possible and were provided with digital dictaphones for this purpose.

Recruitment for patient interviews was conducted purposively with participants chosen for maximum diversity including men and women, different ages, medical and surgical arms of the trial, and from different sites. Selected patients who consented to be contacted by the research team, were invited by one of the qualitative researchers (CM) by telephone to take part in an interview. We invited participants for interview as soon as possible after they had been randomised, in order to obtain their recent recollections not only of being recruited to the MACRO trial but also their reactions to learning of their treatment allocation. We were not able to interview any patient who declined the MACRO trial as none had given consent to be contacted for interview (patients who declined MACRO often declined taking part in the MCS). Recruitment for patient interviews took place between January 2019 and June 2019.

Recruitment for recruiter interviews was inclusive with all 17 recruiters on the MACRO trial invited by email to take part by the qualitative research team, as well as the Trial Manager. Sampling and interviews for both groups were conducted sequentially. We aimed to find the optimal point for interview when the recruiter felt that they had conducted sufficient recruitment conversations to be able to give in-depth feedback and reflection on the process, whilst also enabling recruitment issues to be identified early enough in the pilot trial to be able to address them effectively prior to the funder’s checkpoint report (and hence progression to the main trial). Recruiter interviews were conducted by one of the qualitative researchers (CM) between March 2019 and June 2019.

### Qualitative researchers

The study was conducted by two female post-doctoral qualitative researchers (CM and JV) with > 10 years research experience, based in a medical research department in a UK university. CM conducted the interviews, CM and JV were both responsible for data analysis and JV conducted the feedback/discussion sessions with recruiter teams. CM had no contact with recruiters or patients prior to the MCS. JV had worked with several recruiters previously but had no contact with trial patients.

### Consent procedures

Patients received the MCS patient information sheet from their clinical team. Participants gave informed verbal or written consent prior to their consultation being recorded but then provided written consent before the recording could be included in the MCS. Patients also had the option of taking part in an interview. All recruiters gave written informed consent for audio-recording and telephone interview prior to the start of the MCS.

### Data collection

#### Audio-recorded recruitment conversations

Face-to-face and telephone recruitment conversations were recorded by recruiters using a hand-held digital audio-recorder. Audio-recordings were transferred securely using an encrypted online data transfer programme and stored on a university computer according to data protection requirements of the University of Southampton. We asked recruiters to keep the dictaphone switched on throughout the consultation, rather than switch it on and off when they perceived the conversation to be relevant to recruitment communication. For this reason, the recordings captured not only content directly relevant to the MCS, but also less relevant material, such as clinicians performing nasoendoscopy examinations or planning future appointments with the patient. Audio-recordings were transcribed verbatim for all sections of the recording which were of relevance to the MCS (any content about the MACRO trial or research generally, conversation about treatments, symptoms or clinical history). For sections of the recording which were not directly relevant to the MCS study (e.g. doctor conducting physical examinations, making appointments with patient, getting the patient to fill out paperwork), these activities were summarised on the transcript by the transcriber, together with the duration. Members of the qualitative research team checked each transcript against the full original audio-recording to ensure that all information relevant to the MCS had been transcribed and that any non-relevant activities had been summarised accurately.

#### Interviews with patients and recruiters

All interviews were conducted by telephone by the qualitative researcher, CM, between January 2019 and June 2019. We developed the semi-structured interview schedules based on our early qualitative work with CRS patients and clinicians [17, 18] with the help of patient and public contributors, researchers and clinicians and informed by published qualitative recruitment investigations [6, 24, 25]. Field notes were kept to document contextual details. The original interview schedule was refined through pilot interviews. Examples of interview questions can be found in Table 3: Interview questions for patients, and Table 4: examples of interview questions for recruiters. No repeat interviews were conducted. All interview audio-recordings were transcribed verbatim in full with any person-identifiable details removed or replaced with codes for anonymity.

**Table 3:** Interview questions for patients

**Examples of questions included in the participant interview**	
- What is your experience of having CRS? (when did it start, duration, symptoms, previous treatments)	
- How did you first hear about the MACRO trial (first contact and impressions,	
- What information were you given about the MACRO trial (including trial purpose, design, interventions, randomisation possible risks and side effects, follow-up)	
- Check participant’s understanding of key areas (randomisation procedures, surgery, medication, placebo)	
- Check whether participant felt their recruiter had a preferred treatment or whether interventions were presented in a balanced manner (equipoise)	
- How did you feel about being invited to take part?	
- What were your reasons for deciding whether (or not) to take part.	
- Did you have any concerns or queries about the trial?	
- How were these answered by the recruiter/research nurse?	
- How did you find the experience of being recruited/screened for MACRO and were there any ways this could be improved for other patients in the future?	
- Feedback on the participant information sheet and other written materials	
- Feedback on any other ways to improve patients’ experience on the MACRO trial.

**Table 4:** Interview questions for recruiters

**Examples of questions included in the recruiter interview**	
- When did recruitment start at your site and how has it been so far?	
**Recruitment pathway**	
- Could you talk me through the usual patient pathway for your site? (e.g. how are patients referred to your site? Identifying eligible patients? Who first introduces the MACRO trial to patients and how do they do this? Who assesses the patient for eligibility?	
**Introducing/explaining the trial.**	
- Please could you tell me how you introduce and explain the trial to patients?	
- How do you explain to patients what will be involved?	
- How do you explain the three different treatment arms? What order?	
- Placebo?	
- What sort of questions have patients asked so far and how do you respond?	
** Randomisation**	
- How do you explain to patients how their treatment will be decided?	
- How easy do you think is it for patients to understand the concept of randomisation?	
- Have you had any doubts about whether a patient really understands randomisation?	
**Uncertainty**	
The rationale for the study is that we don’t know what the best treatment is for CRS.	
- How have you found that patients react to the idea that medical science does not know what the best treatment is? How do you respond to this? How does the uncertainty make you feel as a clinician?	
- Have any patients expressed a preference for a particular treatment? Which and why?	
- Can you talk me though how you respond if a patient expresses a preference?	
- Do you ever have a feeling during an appointment that a patient should really have one treatment rather than another? How do you handle that?	
**Reasons for declining participation in the MACRO trial.**	
- What reasons have patients given for not wanting to take part?	
- How do you respond to patients not wanting to take part?	
- What do you see as the main difficulties patients have with the trial?	
**Learning and training**	
- Have there been any particular learning points so far?	
- What advice would you give to new sites about what might enhance recruitment to the MACRO trial?	
Could you describe any additional training or information which might help your team with recruitment at your site?	

## Analysis

Transcripts were checked for accuracy by CM and uploaded onto Nvivo 12 data management software. We used thematic analysis [26] to analyse the datasets in parallel with data collection and used the emerging findings to refine interview schedules. CM acted as the first coder for the transcripts, using Nvivo 12 software. The coding framework was checked for consistency and authenticity against transcripts by JV. Within our analysis, we compared data across data types (i.e. patient interviews, recruiter interviews and audio-recordings of recruitment conversations). We had a small amount of paired data (i.e. matched patient interview and audio-recording of the recruitment consultation) where we could conduct within-case analysis, comparing the recruiter-patient interaction with patient understanding of some of the key themes (e.g. treatment allocation, equipoise). However, there was not enough data to conduct a full paired analysis. We analysed each dataset (i.e. patient interviews, recruiter interviews and audio-recordings of recruitment conversations) individually through a process of coding and refining of concepts. Then, subsequently through an iterative process, we compared these concepts, moving back and forth between the different datasets and emerging themes, to develop a thorough explanation of the data sets.

Within the analysis, JV and CM compared quantitative screening log data against qualitative interview and recruitment conversation data, looking for links, patterns, consistencies or discrepancies, using these to test and refine our initial findings, and seek explanations which were robust and consistent with data across all datasets (triangulation) [7]. In this iterative process, we looked for common patterns within and between datasets, as well as exploring whether there were issues or patterns specific to individual recruiters or sites [7].

CM and JV held regular team data sessions to rigorously review the emerging themes and we compared the final themes with the individual analyses to ensure they accurately represented the individual findings [27].

### Reporting back to the recruiters, TMG and funders

As the analysis progressed, key findings were reported back to recruiters each month in the form of ‘Tips for recruiters’ in the MACRO newsletter. Four months into the 6-month pilot phase, preliminary findings were reported back to the Chief Investigators, the Programme Management Group (PMG) and as a written checkpoint report to the funder. Individual transcripts were not returned to participants for comment, but the end of the 6-month pilot phase, individual feedback was provided to each site as a written summary, followed up by site meetings to discuss the findings with teams of recruiters.

The written summary comprised a report of general findings from the MACRO conversation study, together with individualised feedback. The general findings included (i) raising awareness of the MACRO trial, (ii) practicalities of the MACRO recruitment visit, and (iii) approaching patients. In each case, we presented the challenges to recruitment or difficulties maintaining equipoise uncovered and provided suggestions about how they could be addressed. Individual site feedback included a summary of the positive aspects of trial conduct and any difficulties encountered, in order to make a plan for effective recruitment going forward. Individual feedback was conducted in a sensitive manner.

The findings of the study were also used to develop a structured recruitment consultation guide for recruiters in the main trial. The guide included three phases: phase 1: starting the conversation (introductions, agenda setting), phase 2: about the trial (explaining the trial treatments, randomisation and trial visits), and phase 3: bringing the consultation to a close (eliciting concerns, decision about taking part).

Key findings were also presented at the MACRO trial investigator meeting and incorporated into training materials for new sites.

## Findings

### Participation in the MCS

Five of the six pilot sites participated in the MCS. One site experienced delays in set-up and was unable to be included.

### Patient participants

Twenty participants, all of whom had accepted participation in the MACRO trial, were purposefully sampled and invited for interview by the researcher CM between January 2019 and June 2020. None declined interview but one was unable to take part within the study dates due to illness, giving 19 patient participants in total. There were 13 men and 9 women, a gender ratio similar to that found in the MACRO pilot trial overall. A cross-sectional study of 1 year of sinus surgery in England found that two thirds of patients receiving polypectomy for CRS were male [28]. Whilst the reasons for gender differences in prevalence and treatment of CRS are complex [29] and beyond the remit of this paper, this suggests that the gender ratio for our sample was appropriate.

Their mean age was 53 years. Nine participants had been randomised to surgery and nine to the medical arm of the trial, with one participant still waiting to hear their allocation. Table 5 summarises participant characteristics. The mean duration of interviews was 29 min (range 18–60 min).

**Table 5:** Characteristics of interviewed patients

Total number of interviewed patients	*n* = 19
Male	*n* = 13
Female	*n* = 9
Age (median, range)	53 years (27–82)
Intervention allocation	
Surgery	*n* = 9
Medication (antibiotic or placebo)	*n* = 9
Not yet randomised to treatment	*n* = 1
Total number of interviewees recruited from 5 pilot sites	*n* = 19
Site 1	*n* = 4
Site 2	*n* = 3
Site 3	*n* = 4
Site 4	*n* = 4
Site 5	*n* = 4

All interviewed patients had accepted participation in the MACRO trial

### Recruiters

Seventeen recruiters were invited to take part from five participating sites, of which 15 were interviewed, including all of the principal investigators. Two recruiters (one nurse and one surgeon) expressed willingness to be interviewed but in the event, it was not possible to arrange interviews before the study end date due to their limited availability. All sites provided at least two recruiters for interview. The interviewed recruiters consisted of seven ENT surgeons, one consultant ENT physician, five research nurses and two research assistants. We also interviewed the MACRO trial manager giving a total of 16 recruiter/research staff interviews. Table 6 provides recruiter characteristics. The mean duration of interviews was 33 min (range 19–62 min).

**Table 6:** Characteristics of interviewed recruiters

Total number of recruiters	*n* = 15
Male recruiters	*n* = 7
Female recruiters	*n* = 8
Profession/role on MACRO Trial	
Surgeon	*n* = 7
Consultant physician	*n* = 1
Research nurse	*n* = 5
Research technician	*n* = 2
Number of recruiters interviewed from each recruiting site	
Site 1	*n* = 3
Site 2	*n* = 2
Site 3	*n* = 4
Site 4	*n* = 4
Site 5	*n* = 2

### Audio-recorded recruitment conversations

Four sites provided 61 recruitment conversations. Of these, 57 were face to face clinic appointments with the recruiter and four were telephone calls. One site reported logistical issues and was unable to record any of their consultations. The mean duration of the recordings was 11.5 min. The recruiters were surgeons (24 recordings), an ENT surgeon, ENT consultant physician and research nurse working together in tandem with all three recruiters at each appointment (20 recordings), research nurses or research assistants (15 recordings) and a specialist registrar (2 recordings). Table 7 summarises the recruitment recording data.

**Table 7:** Recorded recruitment conversation characteristics

**Number of recorded recruitment conversations**	*n* = 61
**Duration (range)**	11.5 min (3 min–1 h 29 min)
**Gender of patient participants**	15 women, 46 men
**Face-to-face consultations in clinic**	*n* = 57
**Telephone conversations**	*n* = 4
**Profession/role of recruiter(s) conducting recruitment conversation**	
Surgeon	*n* = 23
Research nurse or research assistant/technician	*n* = 15
Surgical registrar	*n* = 3
More than one member of research team working together (e.g. surgeon, consultant physician + research nurse or registrar + research nurse or surgeon + registrar)	*n* = 20
**Number of recordings for each trial site**	
Site 1	*n* = 24
Site 2	*n* = 0
Site 3	*n* = 20
Site 4	*n* = 15
Site 5	*n* = 2

We experienced difficulties in obtaining a representative data spread for recorded recruitment conversations across all recruiters. Generally, recruitment conversations tended to be recorded by one recruiter (or doctor/nurse recruiter team) at each site, rather than by all recruiters at the site. Overall, it was noticeable that junior doctors and research nurses were under-represented within the recording data.

### Screening logs

Screening logs were completed by all sites. The QRI recommends the use of the SEAR framework for analysing screening log data (recording numbers of Screened, Eligible, Approached and Randomised, as well as reasons given for declining participation) [30]. However, the patient pathway to MACRO was complex, with screening at multiple time-points, including both before and after the patient was approached for the trial. In addition, some patients who may be ineligible at one time point (e.g. due to excluded medication) subsequently become eligible at future visits. For this reason, the SEAR framework, which depicts the four stages of recruitment in a linear sequential progression, was not a natural fit for our data. However, our analysis of the data from our screening logs did demonstrate that of the 259 patients who were screened for eligibility (through hospital notes, outpatient appointments and clinical screening), 91 were found to be eligible and 65 subsequently agreed to be randomised into the MACRO trial. Of the eligible patients, 65/91 (71%) accepted participation, which appears a relatively high proportion of potential participants. This may in part be due to the nature of the recruitment pathway, in which a patient had to give signed consent to the MACRO trial before they could receive the more intensive aspects of screening, such as ECG and CT scan. This meant that only patients with a potential interest in becoming MACRO participants were fully screened. However, this data provides some indirect confirmation that recruiters were providing well-balanced information to patients throughout the recruitment pathway, demonstrating triangulation of screening log data with qualitative data.

The most common reason stated for declining participation was treatment preference. Table 8 summarises reasons given for declining participation and this will be discussed further in the ‘Themes’ section of the findings.

**Table 8:** Reasons stated by patients for declining participation in the MACRO trial

Reason stated for declining trial		
**Treatment preference**	***n*** **= 29**	
	**Subcategories**	
	Wanted surgery	7
	Did not want surgery	7
	Wanted antibiotics	1
	Did not want antibiotics	1
	Did not want placebo	4
	Did not want intranasal medication	1
	Wanted oral steroids	1
	Wanted to choose own treatment but preferred treatment not stated	7
**Other reasons**	***N*** **= 7**	
	Time commitment involved	4
	Geographical distance for visits	1
	Concerns about data protection	1
	Did not wish to be on a trial but detail reasons not stated	1

### Themes

Four key themes emerged as potential issues impacting recruitment in the pilot phase. These themes are presented in turn illustrated by anonymised verbatim quotes.

1. *Planning ahead*: the importance of good preparation
2. *Building awareness*: engaging the wider network
3. *Communicating effectively*: getting the message across
4. *Understanding patient motivation:* preferences and expectations

### Theme 1. Planning ahead: the importance of good preparation

Recruiters described the importance of good preparation and organisation for the complex range of tasks needed for the MACRO trial, particularly the number of screening tests and baseline measures required for each patient. The role of research nurses emerged as essential to ensure smooth flow so that a multiplicity of tasks fitted together into a cohesive process. Research nurse time and availability emerged as a key rate-limiting factor for many sites on how quickly each site could get the MACRO trial ‘up and running’ and/or how many patients could be recruited into the trial.

The [research nurse] has transformed our research just in the past… She only been working here for three months… she’s amazingly proactive and really made things a whole lot easier.. (Interview with Recruiter 5, surgeon)

The MACRO trial required engaging with other hospital specialities and departments, as well as numerous complex screening tests and assessments. Local logistical difficulties caused early delays in set up and recruitment to the MACRO trial for most of the sites.

Just that forward planning really and thinking about recruitment to the trial before they’re actually quite ready to be recruited in the trial, because you want to go and get them ready at the next step rather than creating another hoop later on down the line. (Interview with Recruiter 1, surgeon)

Recruiters described starting the MACRO trial as a ‘steep learning curve’, which was becoming easier as the component stages became more familiar. This view appeared to be common to all research nurses/research technicians and most surgeons or physicians that we interviewed. Two research nurses recommended conducting a ‘mock run’ prior to the first patient screening visit to check for any difficulties.

Mutual encouragement and good team communication were described by most recruiters as important in maintaining motivation and effective teamwork.

I think it’s all about motivation. I think we should have a meeting somewhere along the line to all meet up personally, to discuss what we’ve done, what we’re doing, pat ourselves on the back and motivate each other. (Interview with Recruiter 4, surgeon, site 3)

In summary, good forward planning was required to achieve flow in the recruitment pathway. Research nurses played a central role both in communicating with patients and dealing with logistical issues and having sufficient research nurse time available for the trial was an essential pre-requisite for successful recruitment.

### Theme 2. Building awareness: engaging the wider network

Availability of eligible patients was perceived by recruiters as one of the main limiting factors for recruitment. Gaining support for the MACRO trial among the wider network of clinicians in primary and secondary care was seen as a crucial factor in addressing this issue.

#### GPs and primary care

In some areas, recruiters reported that local NHS initiatives encouraging GPs to manage more CRS patients in primary care had significantly reduced the number of referrals of uncomplicated CRS patients. This had resulted in a reduction of available patients to approach for the MACRO trial. In addition, local prescribing recommendations for CRS in primary care, such as oral steroids and longer-term antibiotics, had resulted in some patients being ineligible for the MACRO trial on presentation in secondary care.

Raising awareness of the MACRO trial through presentations to GP meetings and/or informal networking was found to be successful in some areas. However, recruiters from other sites reported that communicating directly with GPs had little effect if this request conflicted with local referral and prescribing policy.

I guess the only way is try to speak to the CCGs so that they are aware that this is what you’re doing. Maybe disseminating things at a much higher level might be much more useful because the commissioning groups are the ones putting pressure on the GPs not to refer patients to secondary care and to institute certain treatments ahead of time. (Interview with Recruiter 9, surgeon)

Recruiters believed that higher-level dialogue with CCGs to gain support for MACRO at an organisational level might provide a more cohesive and integrated situation, especially if combined with networking with local GPs. Two recruiters reported that they were planning such discussions.

#### Hospital colleagues

Recruiters reported that potentially eligible patients were often first seen by other clinicians at the hospital who were not on the MACRO team, including trainees. Recruiters perceived that clinical colleagues could either facilitate or hinder MACRO recruitment directly (by alerting or not alerting recruiters to an eligible patient) or indirectly (by expressing strong treatment preferences or recommendations to a patient which could inadvertently create psychological barriers to their willingness to taking part).

I think I had underestimated the importance of getting the trainees onside … They’re seeing half the patients, at least if patients aren’t all seen by me and they are all seen by other members of the team. It’s important that they are all on the same page or we’re going to lose any patient coming through their hands. (Interview with Recruiter 7, surgeon)

To address this issue, recruiters described efforts they had made to network with hospital colleagues, both junior doctors and senior colleagues. Strategies described included presenting at departmental meetings and, conducting training sessions on communicating different treatments in a balanced manner (equipoise). Some recruiters had encouraged junior colleagues to do the online GRANULE training for recruiters, which includes training in equipoise [31]. Two senior recruiters had encouraged junior members of their team to join the NIHR Associate Principal Investigator scheme [32], which enables junior doctors to join a research team as an Associate PI. As several recruiters noted, this not only provided junior doctors with valuable experience and a useful addition to their Curriculum Vitae, but also provided the MACRO trial with early career, enthusiastic recruiters who could elicit the support of their peers through face to face and online forums for junior doctors. In the following quote, a junior doctor who had recently joined the MACRO trial as an API described his initiatives to raise awareness of the MACRO trial among colleagues.

Speaking to colleagues in the outpatients, talking about the study, asking them to consider whether a patient might be suitable, if they’ve got CRS and may be seeking further treatment. I’ve also been using our trainee WhatsApp group to basically achieve the same thing, and on Friday we have a clinical governance meeting where I’m going to be giving a short presentation to the whole Trust about the study as well. (Interview with Recruiter 13)

The key learning points from this theme were the need to get the wider network of clinical colleagues ‘on board’ including GPs, CCGs and senior and junior hospital colleagues to improve the flow of potentially eligible patients for trial.

### Theme 3. Communicating effectively: getting the message across

A key aspect of this study was to explore and understand how recruiters present the trial to patients in order to identify any issues or learning points.

The recruitment conversation recordings provided the best level of evidence for assessing recruiter communication. For recruiters who sent recordings, our analysis of these recordings suggested that whilst presentation styles differed, recruiters generally presented the study clearly and accurately. The language used in presenting the trial appeared consistent with recommendations given in the pre-trial training and guidance sheets.

The premise of the trial is that we’ve got two treatments under investigation in the trial; one is long-term antibiotics for 12 weeks, one is sinus surgery and the reason we’re doing the trial is we don’t know which is the best one in the longer term, to offer to patients. So we have to sort of do a trial where there’s an equal chance of getting them. (Audio-recording of recruitment conversation, Recruiter 1, patient participant 2)

In this quote, the recruiter uses simple direct language as recommended by the recruiter training to communicate equipoise (‘we don’t know which one is best’) and to explain randomisation (‘equal change of getting them’). The recruiter has avoided any use of metaphors which could be misunderstood or descriptive language that implies that one treatment is better than another.

For those recruiters who did not send recruitment conversation recordings, we were unable to ascertain directly how their communication compared to their training guidelines. However, we were able to conduct some indirect assessment of the effectiveness of their communication by questioning patients about how well they understood different aspects of the trial.

Patient interview data indicated that patients had good comprehension of the purpose and design of the trial and the process of randomisation. They also showed good understanding of the placebo and surgical arms of the trial. However, several patients appeared uncertain about the medication arm of the trial and seemed unclear about the difference between steroids and antibiotics, especially when the antibiotics were being used for their anti-inflammatory effects, as is the situation in the MACRO trial.

I got it confused because I was on a steroid, so then I thought that was what he meant by antibiotic. That's just my layman’s understanding of medical terms of steroids and antibiotics; they’re just tablets to me. (Interview with Patient participant 6)

As soon as this issue was identified from patient interviews, we used the ‘Tips for Recruiters’ section of the MACRO monthly newsletter for recruiters to highlight the importance of checking that patients understood the medical arm of the trial, especially the difference between steroids and antibiotics used for anti-inflammatory properties. We also raised this issue within team meetings. Subsequent recordings appeared to demonstrate that recruiters were including this in their explanations, as the quotes below demonstrate.

The point of this study, as we’ve spoken about previously, is that we don’t really know what the best treatment for chronic sinusitis is, and whether it is surgical or medical, and the medical for this particular trial is the use of an antibiotic called clarithromycin, which is going to be mainly used for its anti-inflammatory properties as opposed to antibacterial properties. (Audio-recording Recruiter 15, surgeon)

Equipoise (presenting the different trial interventions in a balanced manner) was a key aspect of the pre-trial training for recruiters. Recruiters were encouraged to reflect on their beliefs about the different interventions and to consider whether their use of language might be disclosing any sense of bias.

I think therefore MACRO is a classic example of doing that because you’re balancing surgical and medical treatment. I think that bringing it back to the role of being a surgeon and talking about alternative options for the operation, which inherently as a surgeon you're biased towards offering operations because that’s what you like doing. (Interview with Recruiter 1, surgeon)

Evidence from audio-recorded conversations suggested that equipoise was generally good among those recruiters who sent recordings. In the following quote, a recruiter who is a surgeon explains the rationale for both the surgical and the antibiotic arms of the study, demonstrating a balanced attitude towards both treatments.

If you were in the surgical group, the idea of that intervention is to improve delivery of treatment to the sinuses, so that the nasal steroid for instance gets further into the nose than it would without. If you were in the antibiotic group the idea is that that medication actually reduces the swelling in the sinuses, which is contributing to the discharge you’re getting from your nose. So they are kind of different means of sort of treating a similar problem.

(Audio-recording, recruiter 1, surgeon)

Here, the surgeon provides an explanation of the therapeutic mechanism for both surgery and antibiotics, emphasising the similarity of purpose between the two.

Comparing this with the wording chosen by a medical physician to compare the treatments indicated no discernible bias either for or against surgery, as the quote below demonstrates.

Now—as you know—you can either have surgery for this condition or you can have medical treatment and we think they’re both as good as each other, but we want to find out if that’s really the case.

(Audio-recording, Recruiter 4, medical physician)

In this quote, the physician does not provide explanations of mechanisms but simply states of surgery and antibiotics that ‘we think they’re both as good as each other’ (communicating equipoise), leading on explaining the purpose of the trial.

For those recruiters who did not record their recruitment conversations, we were unable to check equipoise directly. However, as an indirect means of exploring recruiters’ ability to communicate equipoise, our interview schedule for patients included a question about whether the patient felt they could sense whether their recruiter would prefer the patient to receive a particular treatment on the trial. Most participants reported that they felt they did not know which their recruiter preferred, as in the following illustrative quote.

Interviewer question: Did you have any sense, when you were talking to the surgeon whether they rather hoped that you might get one or the other [of the treatments]?

Patient participant: No, they seemed really neutral. (Interview with Patient participant 6)

However, for one patient, a sense of recruiter treatment preference was perceived, even if not explicitly stated. The following quote provides the patient’s own account of the recruitment conversation.

I just got a feeling, I may be wrong but I just got a feeling that they would prefer me to go through the surgery even if I didn’t take part in the trial, I just got the feeling they probably thought, yes, let’s get in there and have a good old dig around (Interview with Patient participant 5)

We were able to review the original audio-recorded recruitment consultation for this patient and examine all places in the consultation where treatment was mentioned. During the main presentation and explanation of the trial, the recruiter used careful wording which appeared to convey good equipoise. However, at a later stage in the consultation, the conversation switched to the recruiter reassuring the patient about how they would be cared for if a medication did not work (this task within medical consultations of managing uncertainties and/or risks is termed ‘safety netting’) [33]. It appeared that during safety-netting, there was an inferred bias that surgery might be the better option. The two quotes illustrate this contrast below.

Explaining the study at the start of the consultation

Recruiter: It’s a horrible condition, I’m sure you’ll agree?

Patient: Yes

Recruiter: and despite all our efforts to treat this, we don’t really know if what we’re using as treatment is the best treatment, if this treatment really even works and that’s why we’re doing this study.

*Safety-netting later in the same consultation*

*Recruiter: And the worst scenario is—that...you know, you get worse—because you do need surgery and if that’s the case, you know, we...will do what’s best for you. So I’m not going to keep you…*

*Patient: Right*

*Recruiter: for years, denying you a treatment that I think might help, if you’re not doing very well.*

(Audio-recording recruiter 5, surgeon, patient 5)

This raised an important training issue to remind recruiters of the need to maintain equipoise, not only in their main presentation of the trial to the patient, but also throughout the consultation. At the same time, it is interesting to note that the patient agreed to take part in the trial, despite their perception that the recruiting surgeon might prefer surgery, suggesting that the safety netting may have provided reassurance or enhanced trust.

In summary, communication among recruiters appeared generally clear and well balanced. Overall, recruitment recording data and patients’ own accounts of their recruitment conversations suggested that presentation style appeared to matter less than trust and rapport in the clinician-patient relationship, providing that information was communicated clearly. The weakest area for patient understanding appeared to be the medication arm of the trial, suggesting that future training for recruiters should highlight the need for checking pharmacological comprehension when talking to patients.

### Theme 4. Understanding patient motivations: preferences and expectations

Patient motivation for taking part in clinical trials, together with reasons for declining, were multifactorial. Exploring patient preferences, hopes and expectations of participating in the MACRO trial helped us to identify possible areas for improved communication, additional training needs or better information sharing.

Data from screening logs showed that among patients who gave a reason for declining to participate, treatment preferences were the most common reason stated. This data is summarised in Table 8. Examining reasons for declining the trial, patient preferences for and against surgery appeared balanced (pro surgery *n* = 7, against surgery *n* = 7). Preference for and against antibiotics was also balanced, though less common (pro antibiotics *n* = 1, against antibiotics *n* = 1). Seven patients stated that they wished to choose their own treatment rather than be randomised, but did not indicate a specific treatment preference. Three patients stated that they did not want to receive a placebo. This reason for declining participation is illustrated below by a quote from the single recruitment conversation recording that was made with a patient who declined the trial.

Patient: I just thought that if I was on the placebo capsule, I would then be another maybe six months without anything, without anything progressing in – in my symptoms, basically.

Recruiter: Okay

Patient: And being on the [clinic] list for probably a couple years now, I just feel that I needed to progress a bit quicker and not take the chance of not getting the surgery or the antibiotics. (Audio-recording of recruitment conversation, Recruiter 1, patient participant 15)

Here, the patient explains the context around why they are unhappy about the possibility of receiving a placebo, in terms of how long they have been already experiencing CRS symptoms and waiting for help and that being randomised to placebo could lead to 6 months more without any improvement. Most of the interviewed patients reported lengthy durations of CRS symptoms (months or years) and for many, this appeared to underpin concerns about the possibility of being randomised to a treatment that might not help.

As shown by Table 8, reasons for declining the trial unrelated to treatment preference were less common, and included geographical distance (*n* = 4), concerns about data protection (*n* = 1) and time commitment involved (*n* = 1).

Whilst we looked for any patterns emerging for between-site or between-recruiter differences, particularly seeking any patterns which might be linked to how recruiters were presenting the trial to patients, the sample within the MACRO pilot trial was too small to determine any meaningful trends. We also looked for any evidence of change over time, for example, whether reasons for declining altered after the feedback we gave to recruiters on recruitment communication but again, the small sample size made it impossible to determine any meaningful patterns. However, we acknowledge that there could be subtle differences which would require a larger data set to detect.

This data from screening logs accorded with the accounts of recruiting clinicians, as recorded in their interviews (an example of triangulating the quantitative and qualitative data). All interviewed recruiters reported that they had found treatment preferences to be a common reason for patients to decline the trial. Furthermore, several recruiters reflected that recruiting a patient with strong preferences might not be in the best interests of the trial, even if the patient were to agree to take part.

If some people have very strong views that they want to go for surgery or very strong views where they want medicine and no surgery, then there would not be… In my view, it wouldn’t be one of those candidates that would actually work well for the trial because with a lot—the follow-ups that you need and everything else, they might not be fully engaged and they might want to switch over halfway through. (Recruiter 9, surgeon)

Patient interviews provided confirmatory evidence that treatment preferences were a major factor for patients in their thinking and decision making about participation, as well as giving us an opportunity to deepen our understanding of this issue from the patient perspective. Whilst all the patients who were interviewed had accepted participation in the MACRO trial, at least half described feelings of conflict between wanting to take part in the trial, and concern that they might be randomised to a non-preferred treatment. Some interviewed patients reported that finding out their treatment allocation had been a relief, giving welcome news that they were to receive their preferred allocation. For others, it had felt like ‘bad news’, but they had managed to rationalise remaining in the trial despite their allocation, for example by reminding themselves that they could still try their preferred treatment after the trial. Relationship with the trial team, especially their doctor, emerged as a major mitigating factor in making patients feel able to remain on the trial even if they were allocated to their non-preferred treatment.

I was like, ‘I don’t really want to be randomised on to the surgery’, but if I was then at least it would be done quicker and there might be some sort of ancillary benefits of being within the trial framework. As it happened, I didn’t get randomised on to the surgery, so I’m either on the placebo or the drug, which was where I wanted to be, and, ideally, I wanted to be on the drug to see whether it would work. (Interview patient participant 1)

In this quote, the patient describes his reluctance to be randomised to surgery but explains his own internal rationale for taking part despite these feelings. He explains this in terms of speed (at least the surgery would happen quickly), and of other benefits of being ‘within the trial framework’. Perceptions that being on the trial might give additional benefits other than the intervention, such as closer monitoring of CRS symptoms and/or a closer relationship with the clinical team were common to most participants.

The hope of a closer relationship with their clinical team appeared to be a major attraction to patients in taking part. This viewpoint was reiterated strongly throughout interviews with patients and recruiters. Research nurses played a crucial role in providing this ongoing support.

It was like being told that people really get followed-up and you have a nurse that is assigned to you that will help you through, you can talk to her at any time. So I felt a bit okay, this is probably a better way to be a patient. You’re more, I don’t know, close to all the team. (Interview patient participant 13)

The importance of the relationship between recruiting clinicians and patients appeared pivotal. Patients described this in terms of trusting not only their clinician’s medical/surgical expertise but also their commitment to the patient’s best interests. As several recruiters reflected, this appeared to create a safe space, in which patients were willing to accept the uncertainties of being randomised to a treatment that neither they nor the doctor will choose, with different perceived risks and benefits.

I think patient trust is the keyword for anything in this trial. Not even for this trial, for any trial. (Recruiter 6, research nurse)

I trust the people at the hospital. I trust their judgement and they’re quite open to discussion about outcomes and so on. They’ve been very open about this whole process, so no, I don’t think I’d have had any concerns. I suppose I might’ve been disappointed if I’d ended up, if I found out several years down the line I’ve been taking a placebo, but hey, that’s just life, isn’t it? (Interview with patient participant 11)

Trust in surgical expertise seemed to be enhanced by clear information and reassurance about surgical techniques. Descriptions of recent advances in endoscopic sinus surgery were particular valued by those patients who were worried about complications, or who had experienced surgery many years ago when surgical procedures were less sophisticated. Several of the patients interviewed described feeling ‘reassured’ and having their minds ‘put to rest’ by detailed factual explanations from their clinician.

They were very good at explaining… both I think the information I was given previously and then the doctor that I saw, were very good at explaining the side effects and the risk factors. Which is always a bit of a worry, but also I’m quite reassured that I would be in good hands, should any complications occur. (Interview with patient participant 18)

Whilst hope of relief from symptoms was reported by all interviewed patients as a reason for taking part, it was interesting to note that this was rarely given as the sole reason, and was more commonly combined with a wish for a closer relationship with their clinical care team and/or a desire to support research which could help CRS patients in the future.

I suppose essentially when it was suggested to me, it was almost, ‘Well, what have I got to lose?’ I can’t see any downside to it, to be honest. If it helps me or even temporarily, it will be good, and if it helps in overall medical research, it’s another good thing. (Interview patient participant 11)

In summary, the main reason for declining participation was treatment preference, though it was interesting to note from the screening log data that preferences for and against surgery were equal in numbers for those patients who declined the trial. Interview data found that the main motivations for patients to participate were hope of relief for symptoms, a closer relationship with their clinical team, and altruism. Future training will encourage recruiters to highlight these points, for example, patients themselves suggested that it might encourage more patients to take part if recruiters might explain in greater detail ways in which trial could help patients in the future, and to highlight more clearly the additional monitoring and ease of contact with the team that an individual would receive as a research patient.

## Discussion

The QRI approach is receiving increasing interest from trialists as a positive strategy for addressing the perennial challenge of recruitment. Our study therefore offers experiences which may be of interest to others considering whether to apply this approach themselves. Overall, we found applying the QRI to be practical, feasible and helpful in terms of identifying recruitment issues.

Recruitment in the pilot phase of the MACRO pilot trial began slowly but accelerated over 6 months, reaching planned targets in time to progress successfully to the main trial. It is not possible to quantify to what extent the MCS study assisted this acceleration since recruitment was affected by many factors. However, the MCS permitted us to identify and explore a number of important barriers and facilitators, which formed the basis of feedback to recruiters not only through site meetings between MCS researchers and recruiters, but also in the ‘Tips for Recruiters’ section in the monthly newsletter emailed to recruiters throughout the MACRO pilot period.

Key facilitators to recruitment included research nurse involvement, which was seen as pivotal to successful recruitment due to the complexity of trial visits and assessments. Engaging the wider network of clinical colleagues emerged as another important factor, ensuring the patient pathway through primary and secondary care would not be detrimental to trial eligibility. Treatment preferences among patients presented a key barrier to recruitment. However, patient concerns about participation could be mitigated by a strong sense of trust in the recruiting clinician. Hope of relief from symptoms was a common motivating factor for taking part. Research participation was perceived by patients both as a means of obtaining a closer relationship with their clinical team, and a means of ‘giving something back’ to the NHS for the benefit of future patients.

Among those recruiters who made recruitment conversation recordings, the trial was generally clearly presented with good equipoise, possibly as a result of good pre-trial training. However, a useful learning point for future training was the need to alert recruiters to the importance of maintaining equipoise throughout the consultation. Patient interviews suggested good understanding of the trial design, randomisation and the surgical arm of the trial. However, some patients appeared less clear about the medication arm of the trial. A clear presentation of medical and surgical treatment options, together with checking patient understanding, had the potential to allay patient concerns and improve understanding.

### Strengths and weaknesses

The MCS incorporated naturalistic data from recordings as well as direct feedback from recruiters and patients. This allowed us to approach the research question from different perspectives [7]. The MCS was planned into the pilot trial from the outset, enabling us to identify barriers to recruitment early, and to hold discussions to explore possible solutions whilst the pilot phase was still under way. Findings were incorporated into reports to the Programme Management Group and contributed to the NIHR checkpoint document which was deemed by the funders to provide justification for progression to the main trial. The MCS team were independent of the Trial Management Team.

The MACRO pilot study provided a limited pool of recruiters, recruited patients and recruitment conversations upon which to draw. For this reason, this study does not claim to have necessarily identified all possible issues or themes (data saturation). However, it was interesting to note that the final interviews elicited very little additional information or insights relevant to the study which had not already emerged from earlier data collection.

Obtaining a representative spread of recruitment conversation recordings proved the most difficult aspect of applying the QRI. A key limitation of the data was that only one consultation was recorded where a patient declined participation and no patients who declined the MACRO Trial agreed to be interviewed for the MCS study. The data therefore sheds more light on why patients did decide to take part, than reasons why patients declined. Whilst we had information from recruiters on reasons why some patients had decided not to take part, there could be factors of which they were not aware. It is possible that the lack of recordings of unsuccessful recruitment conversations was due to recruiter reluctance to record conversations with patients who appeared more negative towards the trial. However, our ongoing conversations with recruiters about this issue throughout the study suggested a more interactive explanation. Research nurses in particular, reported that it could feel uncomfortable, socially sensitive, or even professionally discordant, to be asking a patient for consent to an interview or recording if they were already giving verbal or non-verbal signals of their unwillingness to become involved in research. Since maintaining rapport with patients remains a priority for all good clinicians, this could present a potential challenge for other trialists wishing to apply the QRI. We received this feedback from nurses in conversations held as part of the study management, rather than in their interview, and are therefore unable to provide quotes on this issue. However, it appears to resonate with the work of Donovan et al. on the ‘hidden’ challenge of role conflict faced by clinician recruiters who are trying to balance their role as recruiter with their role of caring for and being sensitive to patient needs and wishes [20].

In theory, it would have been possible for MCS researchers to have attended outpatient clinics to request consent from patients for MCS recordings of their consultations. However, large geographical distances between sites and the small numbers and unpredictable timing of eligible patients per week would make this a very costly strategy relative to the amount of data likely to be collected.

Whilst four of the five recruiting sites provided recruitment conversation recordings, some recruiters provided more than 20 recordings whilst others sent few or none. We received substantially more recordings from ENT consultants with a key role in planning the MACRO trial than from other recruiting doctors or research nurses (see Table 7). This presented us with difficulties in assessing directly whether there might be communication problems affecting other recruiters in the trial. We addressed this as far as possible from the data available by asking patients directly about the information they had received from recruiters and how they felt it was presented. This feedback appeared to suggest that recruiters were generally communicating the trial clearly. We acknowledge, however, the possibility of interviewer effects, where a desire to ‘say the right thing’ might inhibit patients or recruiters from fully expressing their views, although to mitigate this risk we did reassure interviewees that both positive and negative feedback would be equally helpful.

Screening log data provided valuable information on reasons for patients declining the trial, but detecting between-site and between-recruiter differences proved more difficult than anticipated due to the small sample size, the complex recruitment pathway in the MACRO trial, and the limited time over which the pilot study was conducted.

### Comparison with previous literature

The design of this study was based on the QRI work of the University of Bristol QuinteT team [5]. Like the QRI, this study conducted a complex process of triangulation that integrated naturalistic qualitative data from recorded recruitment conversations, qualitative feedback from patient and recruiter interviews, quantitative data from screening logs and mapping recruitment pathways [7]. Whilst some of our experiences appeared similar, some differences also became apparent for findings on the main barriers to recruitment. Within previous QRI work, communication issues such as unclear explanations of trial procedures (e.g. randomisation), lack of equipoise or inadvertently negative language used by recruiters was a key focus for remedial action [14, 34, 35]. In contrast, we found that among those recruiters who provided recordings, communication appeared generally clear and accurate. However, we did identify one example of a need to maintain equipoise during safety netting which raised an important issue about maintaining equipoise in the wider clinical interaction. As Beasant et al. noted, clinician preferences can have detrimental effects on trials other than simply affecting recruitment, including higher drop-out rates and/or patient expectations of treatment outcomes, and hence outcome data [36]. For this reason, finding ways to communicate safety netting without implying treatment bias may be seen to be an important challenge that we intend to take forward into future recruiter training.

Evidence from patient interviews suggested that patients had good understanding of most aspects of the trial, other than a few patients showing pharmacological confusion between antibiotics and steroids. Instead, communication with the wider network of medical colleagues not directly involved in MACRO emerged as a major issue, as did CCG support and logistical issues such as shortage of research nurse time.

These differences may relate to where our study lies on the historical ‘learning curve’ on optimising recruitment. In the MACRO pre-trial training and guidance for recruiters, we highlighted potential communication issues identified by previously published research including equipoise [21, 35] explaining difficult concepts such as randomisation [37, 38] and being aware of potentially negative or unclear language [14, 34]. Some recruiters had also attended courses on recruitment communication covering these and other issues. It was encouraging therefore to find that these areas appeared to be communicated effectively by MACRO recruiters. With communication issues mainly addressed before recruitment began, other rate-limiting factors became apparent, such as lack of equipoise among the wider network of clinicians influencing patient treatment preferences, fewer GP referrals than anticipated and shortage of research nurse time. This in turn related to a need for higher-level NHS support for the trial, including CCGs (see Theme 2: Building awareness). These findings extend previous QRI understanding of communication issues beyond the recruiting clinicians to the wider network of local clinicians and NHS managers and decision-makers. We found that some MACRO sites had been successful in using routine training/networking meetings to increase awareness among clinical colleagues, thereby increasing recruitment. We recommend that addressing this issue as early as possible during the pre-trial set-up phase could enhance recruitment in other clinical trials in the future.

Recruiters suggested that local Clinical Commissioning Group influence on GP referral behaviour might be an important factor in determining how many eligible patients are referred to ENT and therefore the size of the recruitment ‘pool’ available. Again, this finding extends previous QRI findings on identifying barriers within recruitment pathways to include CCGs and primary care. Our findings suggest that if recruitment is to be fully optimised, it may need to consider influencing not just those clinicians directly recruiting for the trial, but the wider organisational context within which they work, maintain professional contacts and receive patient referrals. At the time of writing, the MACRO trial management group were planning how such discussions with local CCGs might best be taken forward in preparation for the MACRO trial. It is not possible at this stage to determine the outcome of this or to what extent it will affect recruitment.

Research nurse capacity was described by all recruiters as a major factor in achieving recruitment. At one high recruiting site, two research nurses were working in tandem on the MACRO trial. They reported that this had made it easier to maintain recruitment flow, since they could provide cover for each other for annual leave and days off, and support one other at particularly busy times. Another high recruiting site had a research nurse whose time was dedicated to the MACRO trial (rather than working on multiple trials). We recognise that such arrangements were not possible for all sites to obtain, but suggest that this issue deserves careful consideration for trialists in the planning stage of any study.

### Implications for practice

Key take home messages for future RCTs include:

- Ensuring sufficient research nurse capacity at each site
- Communicating with the wider network of clinical colleagues in order to ‘get them on board’ as early as possible when setting up trial sites.
- Involving junior doctors as much as possible in recruitment, considering the NIHR Associate Principal Investigator [32] scheme where appropriate.
- Ensuring that recruiters receive pre-trial training in recruitment communication skills, ideally based on previous published work on recruitment communication such as the work of the University of Bristol QuinteT team.
- Reminding recruiters of the importance of maintaining equipoise not only when presenting the trial but also when providing patients with reassurance about what will happen if things do not go to plan on the trial or a treatment does not work (safety netting).
- Recognising the importance of clinician-patient relationships and altruism in patients motivation to take part in trials

The MACRO trial is designed to determine the best treatment for CRS, which could potentially lead to more cost-effective treatment for the condition in the future. Paradoxically, initiatives by some local CCGs to reduce GP referrals for uncomplicated CRS appeared to be currently making recruitment more difficult. This finding highlights the need for high-level collaboration and integration of purpose to ensure that strategically important trials are supported at all levels within the NHS.

The MACRO pre-trial training for recruiters may have helped to encourage clear and accurate presentation, as well as equipoise. However, we are not able to determine the specific effect of this as all MACRO recruiters received similar content. The online NIHR GRANULE training [31] currently offers a free, accessible means of providing training on recruiting to trials, including communicating equipoise. This, combined with trial-specific training for recruiters, may offer a flexible and cost-effective way forward for enhancing recruitment skills for new recruiters.

## Conclusion

The MCS was able to use qualitative research methods to identify a range of issues affecting recruitment for the MACRO trial. This, alongside more traditional trial management techniques, permitted timely and in-depth discussion with recruiters about how recruitment could be maximised at each site. After a slow start, recruitment in the pilot stage of the MACRO trial increased to a rate which met projected targets and led to agreement from the funder to proceed to the main MACRO trial. Although many of the issues raised were specific to this trial, others are more generic and potentially apply to many clinical trials relying on recruitment from routine care settings. The methodology has potential to be useful in other multi-centre clinical trials, and trialists should consider incorporating a QRI component within the pilot phase of planned RCTs in the future.

## Data Availability

The data that support the findings of this study are available on reasonable request from the corresponding author, Dr. Jane Vennik. The data are not publicly available due to containing information that could compromise research participant privacy/consent.
